# Identification of a natural ligand of the hazel allergen Cor a 1

**DOI:** 10.1038/s41598-019-44999-2

**Published:** 2019-06-18

**Authors:** Thessa Jacob, Christian Seutter von Loetzen, Andreas Reuter, Ulrike Lacher, Dirk Schiller, Rainer Schobert, Vera Mahler, Stefan Vieths, Paul Rösch, Kristian Schweimer, Birgitta M. Wöhrl

**Affiliations:** 1 Universität Bayreuth, Lehrstuhl Biopolymere, Bayreuth, Germany; 2 Forschungszentrum für Bio-Makromoleküle, Universitätsstr. 30, D-95447 Bayreuth, Germany; 3 Division of Allergology, Paul-Ehrlich-Institut, D-63225 Langen, Germany; 4 Universität Bayreuth, Lehrstuhl Organische Chemie I, D-95447 Bayreuth, Germany

**Keywords:** Solution-state NMR, Plant molecular biology

## Abstract

Hazelnut is one of the most frequent causes of food allergy. The major hazel allergen in Northern Europe is Cor a 1, which is homologous to the major birch pollen allergen Bet v 1. Both allergens belong to the pathogenesis related class PR-10. We determined the solution structure of Cor a 1.0401 from hazelnut and identified a natural ligand of the protein. The structure reveals the protein fold characteristic for PR-10 family members, which consists of a seven-stranded antiparallel β-sheet, two short α-helices arranged in V-shape and a long C-terminal α-helix encompassing a hydrophobic pocket. However, despite the structural similarities between Cor a 1 and Bet v 1, they bind different ligands. We have shown previously that Bet v 1 binds to quercetin-3-O-sophoroside. Here, we isolated Cor a 1 from hazel pollen and identified the bound ligand, quercetin-3-O-(2“-O-β-D-glucopyranosyl)-β-D-galactopyranoside, by mass spectrometry and nuclear magnetic resonance spectroscopy (NMR). NMR experiments were performed to confirm binding. Remarkably, although it has been shown that PR-10 allergens show promiscuous binding behaviour *in vitro*, we can demonstrate that Cor a 1.0401 and Bet v 1.0101 exhibit highly selective binding for their specific ligand but not for the respective ligand of the other allergen.

## Introduction

Allergy to hazel is very common in Europe^1,2^ and has even been found to be the most frequent cause of IgE-mediated food allergy^3–5^. Cor a 1.04, a Bet v 1 homologous allergen, which belongs to the family of pathogenesis-related plant proteins PR-10^6,7^ is the major hazelnut allergen in Northern Europe^8^. About 53% of people allergic to birch pollen suffer from cross reactivity to Cor a 1.04^9^.

PR-10 proteins are part of the plants’ immune defence and are mostly induced by attack of different pathogens^10,11^ or abiotic stress stimuli^12,13^. However, in certain plant tissues that have higher risks of being attacked by insects, fungi or of being damaged by UV-radiation, PR-10 proteins are expressed constitutively^14^. They are encoded by multiple genes and therefore occur as a mixture of different isoallergens with >67% sequence identity and variants (formerly also called isoforms) thereof, which share a very high sequence identity of >90%^15^.

The molecular role of PR-10 proteins under physiological conditions in different plants remains elusive. Numerous studies on recombinant ligand-free Bet v 1 exist, which show that it binds to a multitude of different ligands *in vitro* like flavonoids, cytokines and fatty acids with dissociation constants in the micromolar range^16–20^.

Different Cor a 1 isoallergens and variants have been identified in hazel pollen as well as in hazelnut and hazel leaves^21^. In hazel pollen four different variants of Cor a 1.01, termed Cor a 1.0101 to Cor a 1.0104, have been detected^22^. Cor a 1.02 and Cor a 1.03 isoallergens can be found in mature hazel leaves^23^. In hazelnut, four different variants of Cor a 1.04, Cor a 1.0401 to Cor a 1.0404 have been identified^7^. Interestingly, Cor a 1.04 variants show a higher sequence identity to Bet v 1.0101 (66–67%) than to Cor a 1.01 variants from hazel pollen (61–65%)^7^.

Although Bet v 1 has been extensively studied biochemically^16,24–27^ as well as immunologically^25,28–31^, the exact physiological role of this protein and its homologs derived from different plants remains elusive.

Typically, the structure of PR-10 proteins consists of a seven-stranded, antiparallel β-sheet and a long, C-terminal α-helix which is enclosed by two shorter helices arranged in V-shape. Those elements encompass a large hydrophobic pocket^32^. Their common structure indicates a more general function, e.g. as storage- or transport-proteins.

To shed light on the physiological role of those proteins, we previously identified the glycosylated flavonoid quercetin-3-O-sophoroside (Q3OS) as a natural ligand of Bet v 1.0101 by co-purification of the protein-ligand-complex from birch pollen^33^. Moreover, we found that different Bet v 1.01 variants show different binding behaviour^34^ and that the binding specificity is driven by the sugar moiety of the ligand^33^.

To investigate whether PR-10 proteins from other plants have identical or similar ligands and ligand binding behaviour, we purified Cor a 1 from hazel pollen in the presence of its ligand. We were able to identify quercetin-3-O-(2″-O-β-D-glucopyranosyl)-β-D-galactopyranoside (Q3O-(Glc)-Gal) as a natural ligand. Compared to Q3OS the only difference between the two ligands is the orientation of the C4 OH group in the first sugar moiety.

Most surprisingly, we can demonstrate that although they are known to show promiscuous ligand binding behaviour, the PR-10 allergens Bet v 1.0101 and Cor a 1.0401 exhibit strong binding specificities only for their own, almost identical ligands. Structure determination of Cor a 1.0401 and binding studies with quercetin, as well as with the ligands Q3OS and Q3O-(Glc)-Gal were performed to analyse the binding specificities.

## Results and Discussion

### Solution structure of Cor a 1.0401

**Figure 1:**
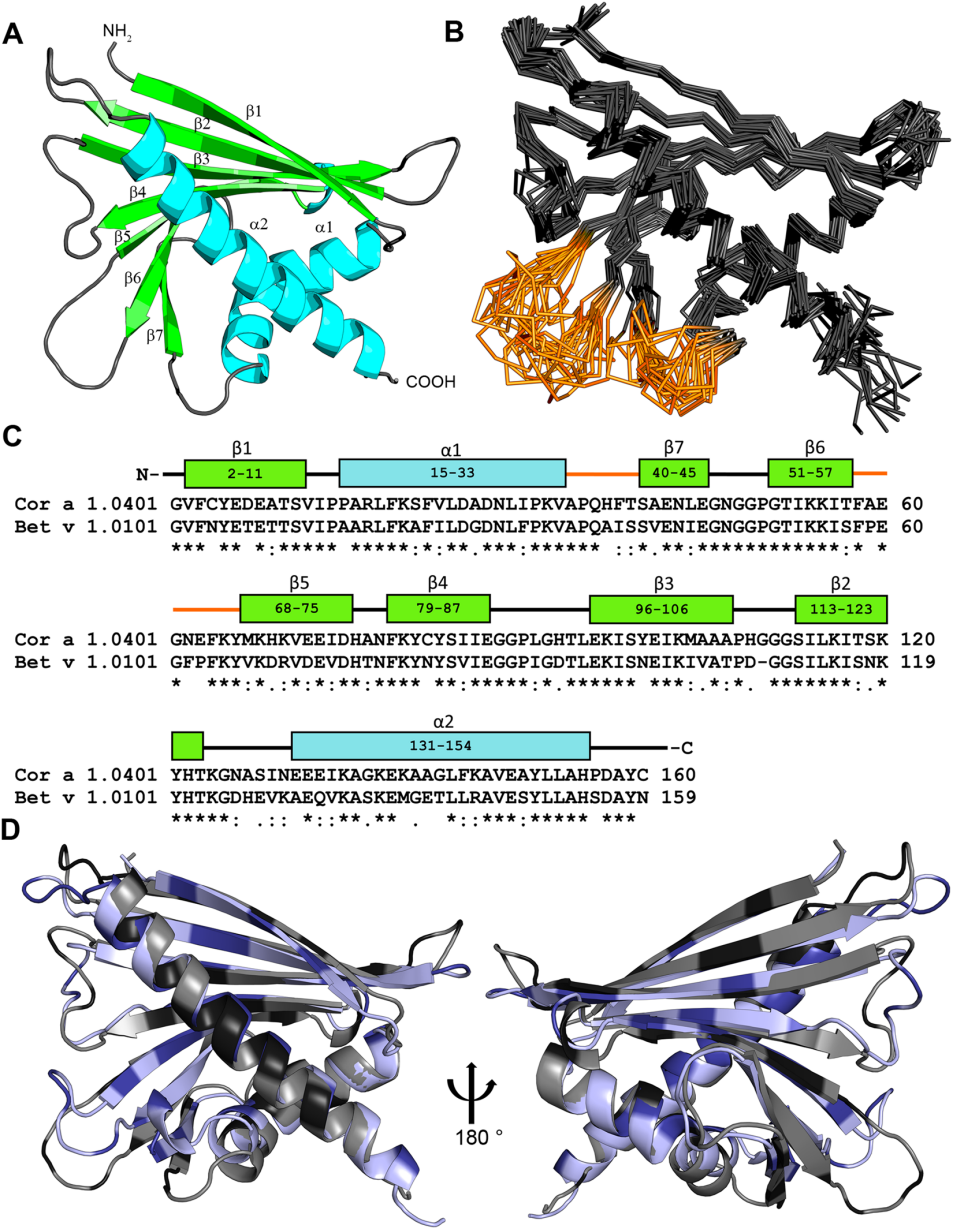
Solution structure of Cor a 1.0401. (**A**) Cartoon representation of the average of the 20 lowest energy solution structures of Cor a 1.0401 (PDB: 6GQ9) (α-helices, turquoise; β-strands, green; loop-regions, grey). (**B**) Backbone overlay of the 20 lowest energy solution structures of Cor a 1.0401, with a backbone rmsd value of 0.93 Å and overall rmsd of 1.26 Å. The loop regions Ala35 - Thr40 (between strand β7 and the two short α-helices) and Thr58 - Met68 (between strand β5 and β6) which did not show resonances are highlighted in brown. (**C**) Amino acid sequence alignment of Cor a 1.0401 and Bet v 1.0101. The α-helices and β strands of Cor a 1.0401 are shown in blue and green, respectively, the two loop regions are highlighted in brown. (**D**) Overlay of the structures of Cor a 1.0401 (grey) and Bet v 1.0101 (light blue) (PDB: 6R3C). Black and dark blue indicate  sequence differences.

To determine the solution structure of Cor a 1 we used a tagless, 160 amino acids long full length construct of the recombinant variant Cor a 1.0401, expressed in *Escherichia coli* (*E. coli*) and performed multidimensional heteronuclear NMR spectroscopy analyses. The spectra exhibited the large dispersion of chemical shifts typical for a well folded globular protein. The structure of Cor a 1.0401 showed the overall fold typical for PR-10 allergens, consisting of a seven-stranded antiparallel β-sheet followed by a long C-terminal helix, which is enclosed by two shorter helices arranged in V-shape that comprise a hydrophobic pocket (Fig. 1A; Table 1). No resonances could be identified in the NMR spectra for residues in the regions Ala35 - Thr40 (between strand β7 and the two short α-helices) and Thr58 - Met68 (between strand β5 and β6) (Fig. 1B,C). These regions consist of loops which, similarly to the strawberry allergen Fra a 1 probably show dynamic behaviour on unfavorable timescales to broaden NMR signals beyond detection^35^. As a consequence of missing signals and corresponding lack of structural restraints a structural definition in this regions was not possible, and these loops differ significantly in the calculated structural ensemble (Fig. 1B). We solved the NMR solution structure of the homologous PR-10 allergen Bet v 1.0101 (PDB: 6R3C; see Supplementary Fig. S1, Table S1) in order to compare the two solution structures (Fig. 1D). The overlay of the Cor a 1.0401 solution structure and the high resolution structure of Bet v 1.0101 reveals the high structural similarity of the two proteins. Despite of the lower number of identified restraints for Cor a 1.0401, the overlay indicates that the structure is sufficiently good to show the overall fold of the protein and to identify its hydrophobic cavity, which is important for ligand binding.

**Table 1:** Solution structure statistics of Cor a 1.0401.

Experimentally derived restraints		
**distance restraints**		
	NOE	845
	intraresidual	1
	sequential	240
	medium range	176
	long range	428
	hydrogen bonds	80
dihedral restraints		208
**restraint violation**		
average distance restraint violation (Å)	0.006020 +/− 0.001643	
distance restraint violation >0.1 Å	1.5 +/− 1.36	
average dihedral restraint violation (°)	0.2134 +/− 0.0452	
dihedral restraint violation >1°	3.00 +/− 1.00	
**deviation from ideal geometry**		
bond length (Å)	0.000583 +/− 0.000061	
bond angle (°)	0.1160 +/− 0.0063	
**coordinate precision** ^**a,b**^		
backbone heavy atoms (Å)	0.93	
all heavy atoms (Å)	1.26	
**Ramachandran plot statistics**^**c**^ (%)	91.5/7.5/0.3/0.7	

^a^The precision of the coordinates is defined as the average atomic root mean square difference between the accepted simulated annealing structures and the corresponding mean structure calculated for the given sequence regions.

^b^Calculated for residues Gly2-Val34, Ser41-Thr58, Lys69-Leu154.

^c^Ramachandran plot statistics are determined by PROCHECK^52^ and noted by most favored/additionally allowed/generously allowed/disallowed.

Additionally, we performed structural overlays with various PR-10 allergens, Pru a v 1 from cherry, Gly m 4 from soy bean and the straberry allergen Fra a 1E, which all exhibit highly similar folds (see Supplementary Fig. S2). Only the length of the loops and the orientation and length of the structural elements are slightly different. One common feature of PR-10 allergens is the so-called glycine-rich loop, which is highly conserved in structure and sequence (see Supplementary Fig. S2), however its function has not been identified yet.

### Identification of the natural ligand of Cor a 1 isolated from pollen

Recently, we were able to identify a natural ligand of the major birch pollen allergen Bet v 1, Q3OS, by co-purification of the allergen in complex with its ligand from pollen^33^. Natural Bet v 1 isolated from pollen consists of a mixture of different Bet v 1 isoallergens and variants thereof. Thus it is difficult to identify which protein(s) bind the ligand. Identification of the natural ligand contributes to the understanding of the function of PR-10 allergens in plants. To determine whether the same ligand can also be found in homologous PR-10 allergens, we purified the natural hazel allergen Cor a 1 from hazel pollen using a similar approach.

**Figure 2:**
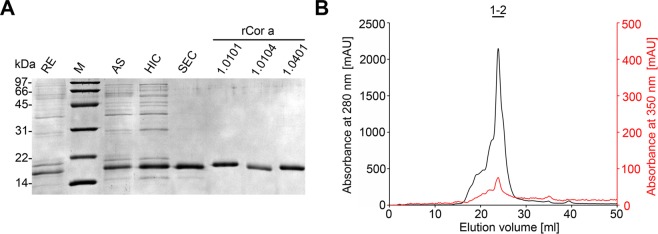
Purification of Cor a 1 from pollen. (**A**) Analysis of the purification procedure of Cor a 1 from hazel pollen by SDS-PAGE (19% gel). The sample after each purification step is shown: RE, raw extract; M, molecular weight marker (low range, Bio-Rad, Munich, Germany), the corresponding masses are indicated on the left; AS, resuspended pellet after precipitation with 100% ammonium sulfate saturation; HIC, pooled fractions after hydrophobic interaction chromatography; SEC, pure Cor a 1 sample after size exclusion chromatography; rCor a 1 represents the three recombinant purified forms Cor a 1.0101, 1.0104 and 1.0401. (**B**) SEC of purified nCor a 1. The absorbance at 280 nm (black line, protein) and 350 nm (red line, ligand) was recorded. Fractions 1-2, were pooled and loaded on the SDS-PA gel shown in **(A)**.

Natural Cor a 1 (nCor a 1) was extracted from mature hazel pollen derived from *Corylus avellana* and purified^33,36^. After each purification step, samples were analyzed by sodium dodecyl sulfate polyacrylamide gel electrophoresis (SDS-PAGE) (Fig. 2A) (see Supplementary Fig. S3). Size exclusion chromatography (SEC) of pooled nCor a 1 fractions collected from a hydrophobic interaction column revealed two absorption maxima at an elution volume of 24 ml, one at 280 nm (protein) and the other one at 350 nm (ligand) (Fig. 2B). Pure SEC fractions were subjected to SDS-PAGE followed by mass spectrometry to identify the proteins present in the peak fractions.

Similarly to nBet v 1, nCor a 1 is composed of different isoallergens and variants and very likely additional ones will be identified. So far, not all known variants of Cor a 1 are detectable by mass spectrometry. We were able to confirm the presence of the variants Cor a 1.0103 and Cor a 1.0104 on the basis of variant specific tryptic peptides. Moreover, we detected Profilin 4, a member of a different allergen family. The proteins identified in nCor a 1 are summarized in Table 2. The detailed data on variant specific peptides and annotated spectra which unambiguously demonstrate the presence of Cor a 1.0103 and Cor a 1.0104 are displayed in Supplementary Table S2 and Fig. S4. Since the variants Cor a 1.0101 and Cor a 1.0102 do not contain unique tryptic peptides they are indistinguishable from Cor a 1.0103 and Cor a 10104. Thus, the presence of these variants can neither be confirmed nor be excluded. However, previous mRNA analyses by Breiteneder *et al*. using RT-PCR indicated their presence in hazel pollen^22^. The isoallergen Cor a 1.0401 could not be detected in our analysis.

**Table 2:** Summary of MS data on natural and recombinant Cor a 1.

internal	Genebank	UniProt	Description	S	SC	E	DP	MP
**(a) purified nCor a 1 in gel**								
PEI127	X70998	Q08407	Cor a 1 0104, Corylus avellana	7180	61.0	5.8	8	1
PEI126	X70997	Q08407	Cor a 1 0103, Corylus avellana	5860	57.2	6.3	7	0
PEI124	X70999	Q08407	Cor a 1 0101, Corylus avellana	—	—	—	—	—
PEI125	X71000	Q08407	Cor a 1 0102, Corylus avellana	—	—	—	—	—
n.a.	n.a.	A4KA45	Profilin 4, Corylus avellana	1723	40.6	7.2	3	0
**(b) rCor a 1.0401 in gel**								
PEI 128	n.a.	n.a.	rCor a 1 0401, Corylus avellana	8489	88.8	6.0	15	1
**(c) rCor a 1.0102 in gel**								
PEI 163	X71000	Q08407	Cor a 1.0102, Corylus avellana	1847	81.9	1.8	18	4
**(d) rCor a 1.0103 in gel**								
PEI 161	X70997	Q08407	Cor a 1.0103, Corylus avellana	2963	81.3	1.3	15	3

n.a. not applicable; S: Score, SC: sequence coverage; E average precursor mass error; DP: number of detected tryptic peptides; MP: number of detected modified peptides.

**Figure 3:**
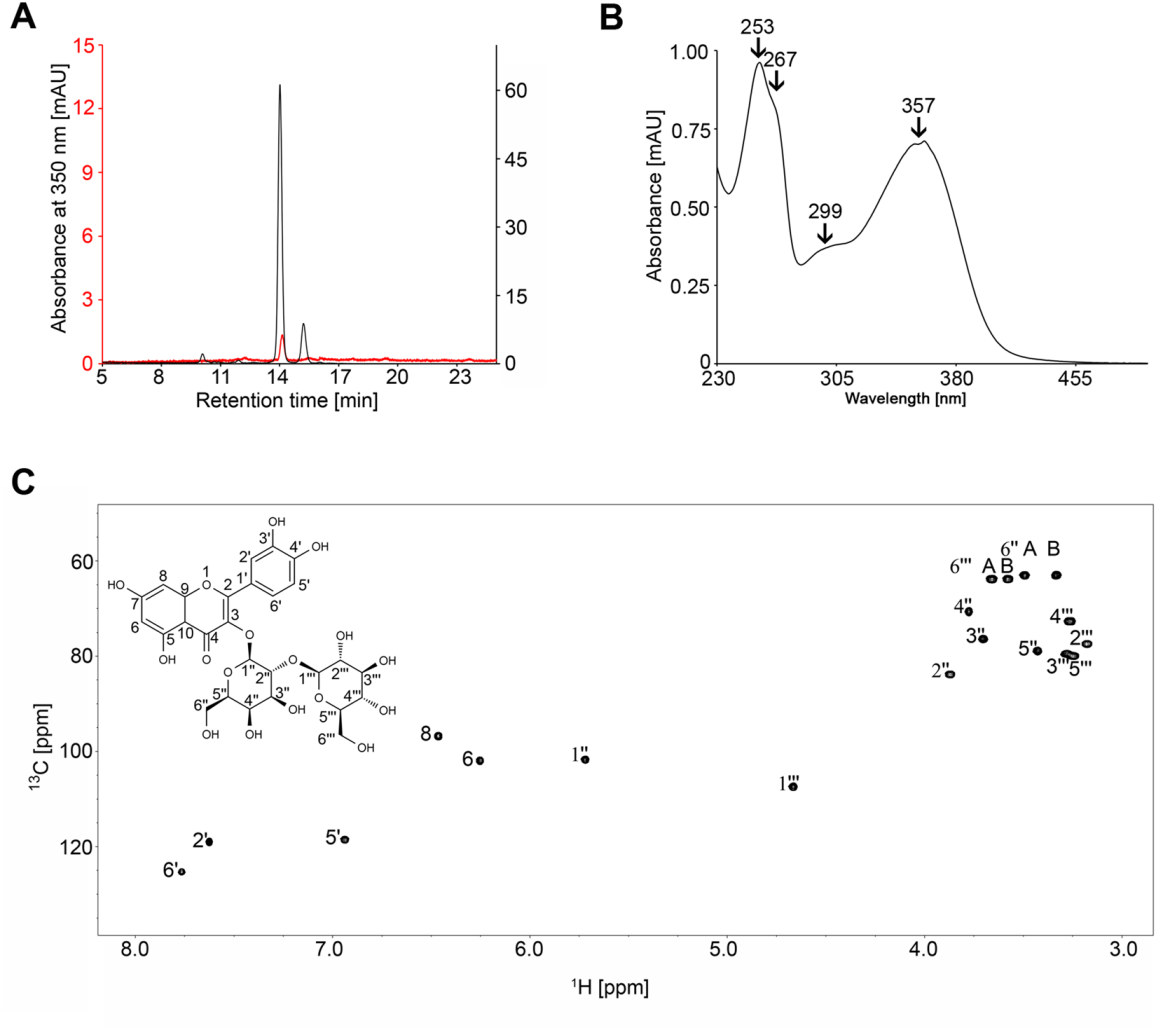
Identification of the natural ligand Q3O-(Glc)-Gal. (**A**) RP-HPLC chromatogram of hazel pollen extract (black line) and the ligand of nCor a 1 isolated from pure SEC fractions (red line) using a C18A column. (**B**) UV/Vis spectrum of the ligand purified from pollen. Absorption maxima at 253 nm, 267 nm, 299 nm, and 357 nm are indicated. (**C**) ^1^H, ^13^C HSQC spectrum of the ligand purified from pollen in d_6_-DMSO. The structure is shown on the top left. A and B indicate the two different hydrogen atoms at positions 6″ and 6″′, respectively.

To identify the bound ligand, SEC fractions containing pure nCor a 1 were lyophilised and extracted with methanol. The extract was analysed both by liquid chromatography / mass spectrometry (LC/MS) (see Supplementary Fig. S5) and reversed phase high performance liquid chromatography (RP-HPLC) (Fig. 3A). The total ion current (TIC) chromatogram (see Supplementary Fig. S5) showed one peak apex at a retention time of 2.4 min exhibiting pseudomolecular ions of m/z = 627.155 [M + H]^+^, which is in good accordance with m/z = 627.156 for the putative molecular formula of the most abundant flavonoid in hazel pollen, Q3O-(Glc)-Gal [C_27_H_30_O_17_ + H]^+^ ^37^. RP-HPLC of the nCor a 1 extract yielded a single peak with a retention time of 14 min (Fig. 3A, red line). To increase the yield of the putative ligand we extracted the ligand directly from hazel pollen, i.e. without purifying the nCor a 1/ligand complex first (Fig. 3A, black line). We performed RP-HPLC and collected the peak fractions with the same retention time, and analyzed them further by UV-Vis spectroscopy (Fig. 3B), mass spectrometry, and NMR spectroscopy (Fig. 3C).

The UV-Vis spectrum of the ligand in methanol exhibited absorption maxima at 256, 269, 299 and 357 nm (Fig. 3B), in excellent agreement with UV-Vis spectra of the glycosylated flavonoid Q3O-(Glc)-Gal in methanol reported previously^37^. The TIC (total ion current) chromatogram of the ligand isolated directly from hazel pollen also confirmed the presence of Q3O-(Glc)-Gal (data not shown).

To further characterize the purified ligand, ^1^H and ^13^C chemical shifts were obtained from a ^1^H, ^13^C heteronuclear single quantum coherence (HSQC) spectrum in deuterated DMSO (d_6_-DMSO) (Fig. 3C). The NMR resonances agree well with published data^37^ and confirmed the identity of this glycosylated flavonoid in the β-glycosidic form. Ligand identification was reproducible using three different pollen batches.

**Figure 4:**
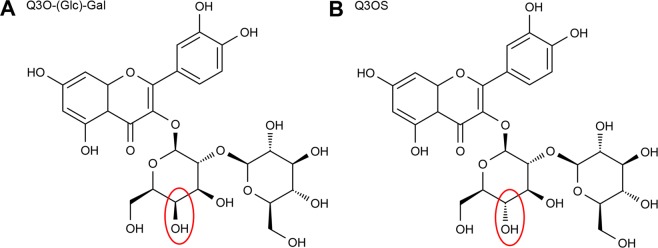
Structural comparison of Q3OS and Q3O-(Glc)-Gal.

Interestingly, this ligand is very similar to the epimeric Bet v 1 ligand Q3OS. The only difference is the orientation of the hydroxyl group at the C4 of the first sugar moiety resulting in a glucose moiety in Q3OS vs a galactose moiety in Q3O-(Glc)-Gal linked to quercetin (Fig. 4).

### Binding of rCor a 1 isoallergens to Q3O-(Glc)-Gal

To learn more about the binding properties of the ligand we analysed its interaction with different rCor a 1 proteins. The high sensitivity of the chemical shift to structural changes makes NMR spectroscopy a powerful tool to investigate ligand binding to a protein as binding of a ligand causes structural changes at least in the binding region. This can be easily observed by comparison of NMR spectra of the protein before and after addition of the ligand.

**Figure 5:**
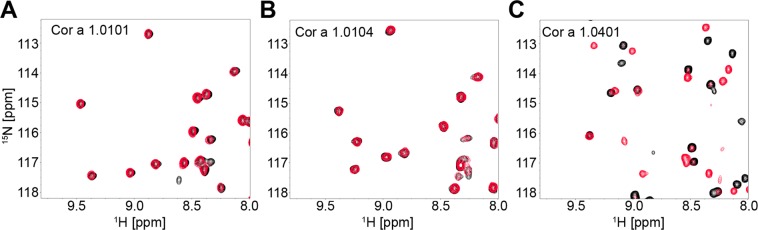
Binding of Q3O-(Glc)-Gal to different Cor a 1 isoallergens. Overlay of two ^1^H-^15^N spectra of 60 µM of Cor a 1 isoallergens in 10 mM sodium phosphate, pH 7.0 in the absence (black) or presence of a ten-fold molar excess of Q3O-(Glc)-Gal (red) recorded at a Bruker Avance 700 MHz spectrometer at 298 K **(A**) Cor a 1.0101, **(B)** Cor a 1.0104, and **(C)** Cor a 1.0401. Upon ligand addition no significant shifts can be observed for Cor a 1.0101 and Cor a 1.0104, implying no or weak binding, whereas with Cor a 1.0401, the signal intensity of many residues decreases and new signals appear, indicating binding.

We decided to further analyse four known Cor a 1 variants previously detected in hazel pollen and one detected in hazel nut^7^. Thus, we purified the ^15^N labelled variants rCor a 1.0101, rCor a 1.0102, rCor a 1.0103, rCor a 1.0104 (pollen), and rCor a 1.0401 (nut) from *E. coli* to perform binding experiments by two-dimensional protein NMR spectroscopy. ^1^H, ^15^N HSQC spectra of the proteins were recorded before and after the addition of a ten-fold molar excess of Q3O-(Glc)-Gal. The spectra of the four Cor a 1 variants from pollen did not show changes even with a ten-fold excess of ligand, indicating no binding. This is exemplary shown for rCor a 1.0101 and rCor a 1.0104, (Fig. 5A,B). In contrast, binding could be observed to rCor a 1.0401 (Fig. 5C) corroborating Q3O-(Glc)-Gal as a ligand.

Cor a 1.0401 was previously shown to be present in hazelnut^7^. To determine whether Cor a 1.0401 is also present in pollen, crude pollen extracts were analyzed by mass spectrometry to avoid loss of individual Cor a 1 isoallergens or variants, which may have occurred during purification of nCor a 1. However, the results matched the ones of purified nCor a 1. We found Cor a 1.0104 and Cor a 1.0103 to be accompanied by Profilin 4 and 5, and several other non allergenic proteins, but not by Cor a 1.0401 (Table S3). Our results indicate that Cor a 1.04 variants are either absent in hazel pollen or they are only present at concentrations below the detection limit. Since rCor a 1.0401 binds Q3O-(Glc)-Gal with high specificity other still unknown Cor a 1 isoallergens may be present in hazel pollen as well as in purified nCor a 1 and bind to the ligand.

Contrariwise, HPLC analyses of hazelnut skin extracts were negative for Q3O-(Glc)-Gal (see Supplementary Fig. S6). This is in line with earlier experiments that introduced 3-O-(2″-O-β-D-glucopyranosyl)-β-D-galactopyranoside conjugates of kaempferol and quercetin as a pollen-specific class of glycosylated flavonoids^38,39^.

### Binding of Q3O-(Glc)-Gal to Cor a 1.0401

**Figure 6:**
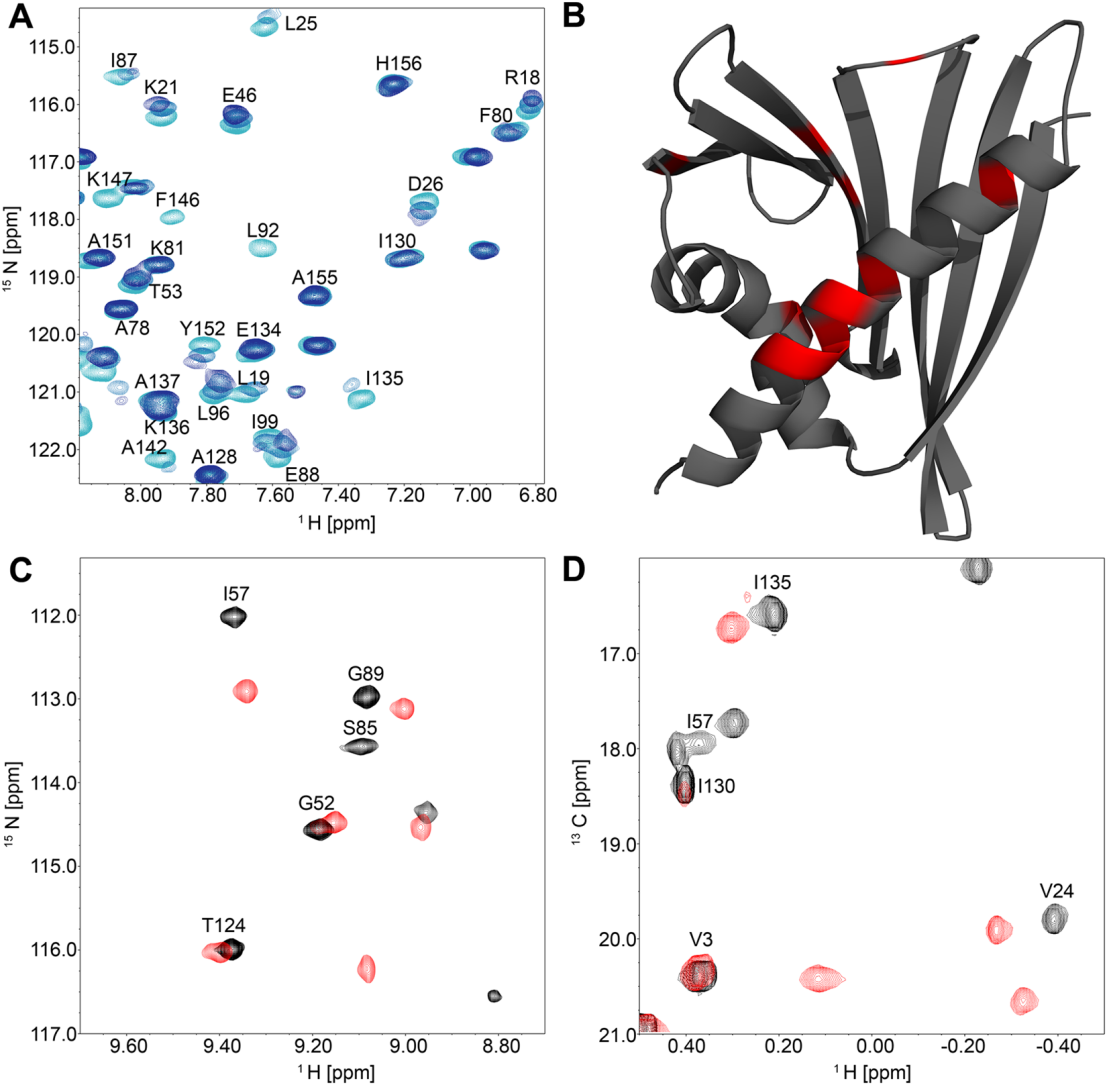
NMR titration of Cor a 1.0401 with quercetin and Q3O-(Glc)-Gal. All experiments were performed with a Bruker Avance 700 MHz spectrometer at 298 K with 60 to 100 μM Cor a 1.0401 uniformly labelled with ^15^N and ^13^C. **(A)** Overlay of three ^1^H-^15^N HSQC spectra of Cor a 1.0401 in the presence of increasing quercetin concentrations, from light to dark blue; ligand to protein ratio: 0, 0.5 and 1. The quercetin stock solution was prepared in d_6_-DMSO to obtain a final DMSO concentration of 2.2% (v/v). **(B)** Cartoon representation of Cor a 1.0401. The amino acids that were strongest affected by quercetin binding are highlighted in red. **(C)** Section of an overlay of two ^1^H, ^15^N HSQC spectra of Cor a 1.0401 in the absence (black) and presence (red) of an 8-fold excess of Q3O-(Glc)-Gal. **(D)** Section of an overlay of two ^1^H, ^13^C HSQC spectra of Cor a 1.0401 in the absence (black) and presence (red) of an 8-fold excess of Q3O-(Glc)-Gal.

To investigate binding of quercetin, which lacks the sugar moiety, to the hydrophobic pocket of Cor a 1.0401, a titration was performed and ^1^H, ^15^N HSQC spectra were recorded after each step (Fig. 6A). While some signals (F146, L92) disappeared, indicating binding in the intermediate or slow exchange regime, others shifted with each titration step (Y152, R18, K21). This is characteristic for binding in the fast exchange resulting in the observation of population-averaged chemical shifts between ligand-bound and unbound protein. The residues which disappeared and therefore were affected most by binding were mapped on the solution structure of Cor a 1.0401 (Fig. 6B). Remarkably, binding of quercetin to Bet v 1.0101 revealed similar, but not identical binding interfaces^33^.

To further investigate binding of Q3O-(Glc)-Gal to Cor a 1.0401, a titration of Q3O-(Glc)-Gal to ^15^N, ^13^C labeled protein was performed and after each step, ^1^H, ^15^N HSQC as well as ^1^H, ^13^C HSQC spectra were recorded. Figure 6C,D show overlays of the ^1^H, ^15^N and ^1^H, ^13^C HSQC spectra, respectively, in the absence of ligand and after the addition of the highest ligand concentration.

During titration, about 80% of all signals disappeared and reappeared somewhere else in the spectrum, which is characteristic for binding in the slow exchange regime. Upon binding of the ligand to the protein, the affected nuclear spins change between at least two states (bound and unbound state) with different chemical shifts. The effect of the exchange on the spectrum depends on the relation between the off rate and the magnitude of the chemical shift difference in Hertz. Observation of spectra in the slow exchange regime implies that the off rate is smaller than the chemical shift difference. A small off rate means slow dissociation of the ligand from the protein, indicating rather strong binding with dissociation constants typically below 5 µM^40^. However, it was not possible to determine exact dissociation constants. As about 80% of all signals were strongly affected by binding, it was also impossible to determine a binding interface. This extreme change in the spectrum could either point towards a structural rearrangement of Cor a 1.0401 upon ligand binding or a rearrangement of the side chains, which influence the chemical environment of the nuclear spins.

### Confirmation of specific ligand binding by two-dimensional NMR experiments

**Figure 7:**
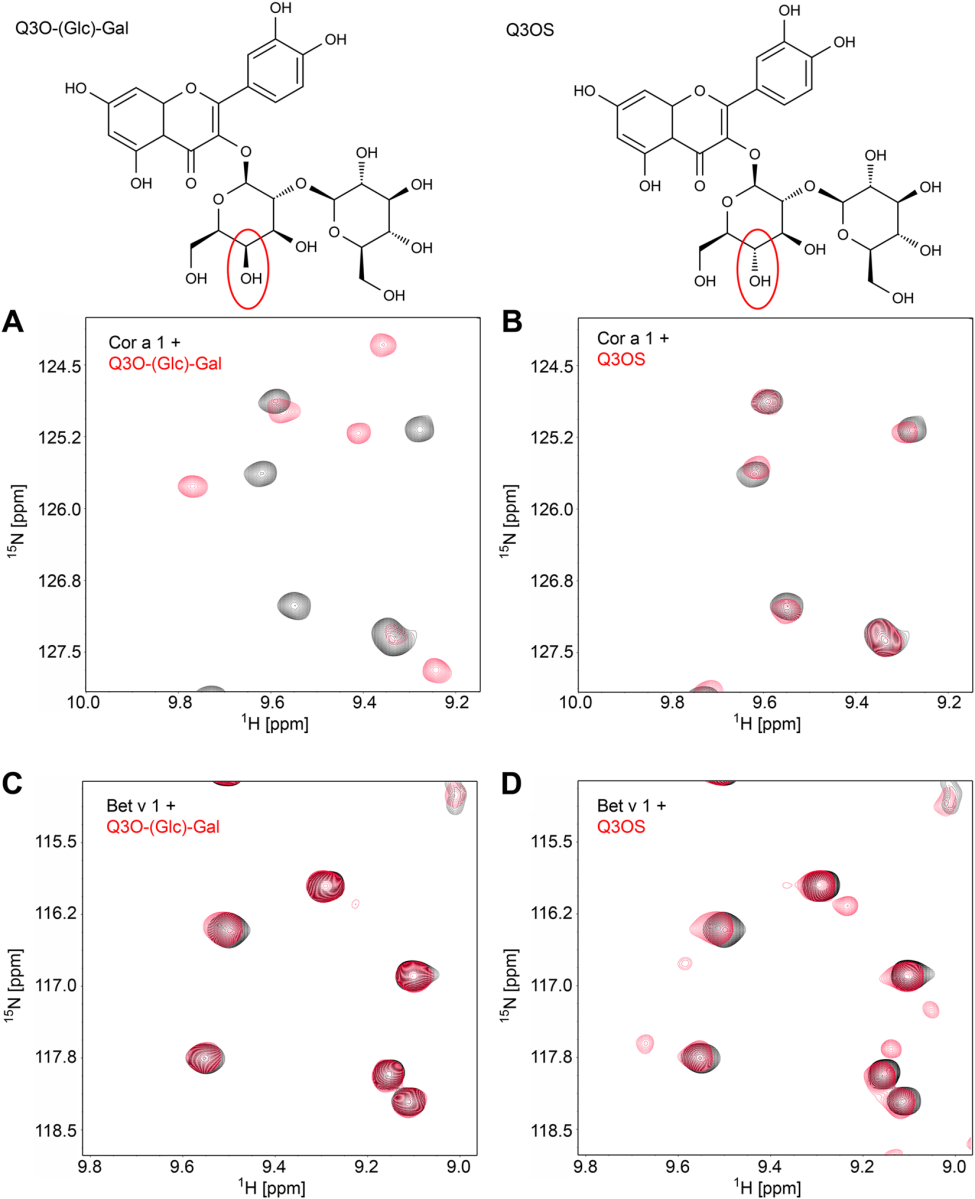
Binding specificity of Bet v 1.0101 and Cor a 1.0401. The structures of Q3O-(Glc)-Gal (left) and Q3OS (right) are shown on top of the panels. Section of overlays of ^1^H-^15^N HSQC spectra of Cor a 1.0401 or Bet v 1.0101 in the absence or presence of ligand: Cor a 1.0401 with **(A)** Q3O-(Glc)-Gal or **(B)** Q3OS; Bet v 1.0101 with **(C)** Q3O-(Glc)-Gal or **(D)** Q3OS.

It is often stated that PR-10-proteins show promiscuous binding behaviour^16,18,19^. To investigate binding specificities, a set of two dimensional NMR-experiments was performed with Bet v 1.0101 and Cor a 1.0401 in the presence and absence of the corresponding natural ligands (Fig. 7).

Most surprisingly, Cor a 1.0401 only binds to Q3O-(Glc)-Gal (Fig. 7A,B), whereas Q3OS binding is specific for Bet v 1.0101 (Fig. 7C,D), even though the amino acid identity of the two proteins is rather high (67.3%) and the only difference between the two ligands is the orientation of the OH-group in the respective sugar moiety (glucose vs. galactose). However, the physiological roles and functions of the different binding specificities remain to be elucidated.

The high binding specificities of Bet v 1 and Cor a 1 variants allow the discrimination between the natural ligand and a highly similar, epimeric ligand of the homologous allergen (Fig. 7). A similar substrate specificity can be observed in glycosyl transferases that are responsible for glycosylation and deglycosylation of flavonoids in pollen^38^. A single point mutation of the UDP-galactose galactosyltransferase from *Aralia cordata* (H374Q) changed the preferential donor from galactose to glucose^41^.

Ligand discrimination might already take place at the entrance to the hydrophobic pocket. The largest opening is located between T58 and Y67. In this region there are three amino acid exchanges between Bet v 1.0101 and Cor a 1,0401, P59A, L62N and P63E, which might play a role in ligand discrimination. However, to explain the molecular basis of the highly selective ligand binding, complex structures of both proteins are needed.

## Conclusions

Despite the high clinical relevance of the hazel allergen Cor a 1, physiological and structural knowledge is scarce. The solution structure of the variant Cor a 1.0401, which we present here, provides the basis for future immunological and physiological studies since Cor a 1.0401 is one of the strong IgE binding variants^7^.

Moreover, we identified Q3O-(Glc)-Gal as a natural ligand of Cor a 1.0401. Our findings demonstrate binding of rCor a 1.0401 usually found in hazelnut to the pollen specific ligand Q3O-(Glc)-Gal. This might be explained by the unusual reproduction biology of hazel. Within 4–7 days after pollination in January and February the pollen grows to the base of the style, which connects the stigma with the ovary. Here, the tip of the tube enters a long resting period of several months. The ovary grows over ca. 5 months until it becomes mature and contains egg cells. The resting sperm becomes activated and after growth of secondary pollen tubes, fertilization takes place. This results in rapid growth of the kernel over a period of 6 weeks^42^.

Glycosylated flavonoids comprise a storage form, whereas the aglycons are functionally active and are indispensable for the formation of the pollen tube^38,43^. Thus, we propose that upon first contact of hazel pollen with the stigma, Q3O-(Glc)-Gal from pollen and Cor a 1.0401 from the stigma might form a complex persisting throughout the maturation of the ovules. Formation of the complex is necessary to prevent premature deglycosylation of Q3O-(Glc)-Gal to quercetin by glycosyltransferases. After maturation, the ligand is released and converted into quercetin, which might then assist the formation of the secondary pollen tube^43,44^. A prerequisite of this hypothesis is, of course, that Cor a 1.0401 is already present in the stigma and is not only expressed during kernel formation. However, this has not been tested yet.

Nevertheless, a yet unidentified Cor a 1 isoallergen bound to Q3O-(Glc)-Gal is present in hazel pollen, since SEC showed that the ligand (627 Da) and nCor a 1 (17 kDa) co-purify in one peak and moreover, we were able to isolate the ligand Q3O-(Glc)-Gal from highly purified nCor a 1. Although peptide mass fingerprint by LC-MS^E^ is a powerful method to identify already known proteins, uncharacterized Cor a 1 isoallergens will not be discovered by this method. It is highly probable that there are more Cor a 1 isoallergens to be found in the future. For Bet v 1, which is the PR-10 allergen investigated most thoroughly, 18 isoallergens have been unambiguously identified so far^45^.

The binding specificity of Bet v 1.0101 and Cor a 1.0401 for their natural ligands, Q3OS and Q3O-(Glc)-Gal, respectively, as well as the fact that the other Cor a 1 variants rCor a 1.0101 and rCor a 1.0104 do not bind Q3O-(Glc)-Gal suggests that despite their high sequence identity and structural similarity, Bet v 1 homologous proteins and variants bind to different ligands and might even fulfil different physiological functions. This might be the reason why a precise function of Bet v 1 homologous proteins could not be identified so far.

## Materials and Methods

### Polyphenols

Quercetin was purchased in analytical grade from Sigma-Aldrich (St. Louis, USA), and Q3OS from Phytolab (Vestenbergsgreuth, Germany). Q3O-(Glc)-Gal was purified from hazel pollen extracts (Allergon, Ängelholm, Sweden). To extract the flavonoids, pollen was dissolved in H_2_O (500 mg dry wt/10 ml H_2_O), stirred at room temperature for 3 h and centrifuged (20 min, 4 °C, 10000 × g). The supernatant was collected and the pellet was redissolved in 10 ml H_2_O, and treated as described above. The procedure was repeated and the extract stirred over night. H_2_O extracts were evaporated to dryness *in vacuo* and the pellets were resuspended in 1 ml of 61% solvent A (2% v/v acidic acid) and 39% solvent B (0.5% v/v acidic acid, 50% v/v acetonitrile), centrifuged and filtered through a nylon filter (45 µm; Phenomenex, Aschaffenburg, Germany). The compounds were purified by HPLC by isocratic elution with 61% solvent A and 39% solvent B using a C18 column (SP 250/21 Nucleosil 100–7; Macherey-Nagel, Düren, Germany) at a flow rate of 10 ml/min. Fractions containing ligands were evaporated to dryness *in vacuo* afterwards. The purified compounds were stored at 4 °C in the dark. Q3O-(Glc)-Gal content was measured by the absorbance at 350 nm, for quantitative calculations the extinction coefficient of Q3OS (ε_350_ = 13500 M^−1^ cm^−1^) was used.

For the analysis of polyphenols from hazelnut skin, the skins were removed from the nuts (type “Barcelona”, origin: USA) with a scalpel and dried overnight at 50 °C. Afterwards, they were shock frozen and grinded using a porcelain mortar. 250 mg of the grinded hazelnut skins was then extracted with 10 ml 100% methanol over night, the extract centrifuged, the supernatant dried *in vacuo* and redissolved in 2 ml of 90% solvent A (2% v/v acidic acid) and 10% solvent B (0.5% v/v acidic acid, 50% v/v acetonitrile). After centrifugation and filtration through a 45 µM nylon filter (Phenomenex, Aschaffenburg, Germany), the extract was analysed by RP-HPLC using a gradient from 10% B to 100% B within 35 min.

### Cloning, expression and protein preparation

Synthetic genes coding for Cor a 1.0101; Cor a 1.0104 (Genescript, Piscataway, New Jersey, USA) and Cor a 1.0401 (optimized for codon-usage in *E. coli*) were cloned via NdeI, BamHI into the expression vector pET11a (Novagen-Merck, Germany). To obtain Cor a 1.0102 and Cor a 1.0103, plasmids pET11a Cor a 1.0104 and pET11a Cor a 1.0101, respectively, were used as templates for site-directed mutagenesis according to the QuickChange Method Cornell iGEM 2012” protocol (http://2012.igem.org/wiki/images/a/a5/Site_Directed_Mutagenesis.pdf).

Gene expression for all unlabelled, ^15^N, and ^15^N, ^13^C labelled allergens was performed as described previously for pET11a_Bet v 1a (Bet v 1.0101)^33^ using (^15^NH_4_)_2_SO_4_ and ^13^C-glucose. An amino acid sequence alignment of the Cor a 1 and Bet v 1 proteins used in this study is shown in Supplementary Fig. S7. The protein bank entry accession numbers and a sequence identity matrix of the proteins are listed in Supplementary Table S4.

### Purification of recombinant proteins

Protein purification for Cor a 1.0101, Cor a 1.0401, and Bet v 1.0101 was performed as described for Bet v 1 a (Bet v 1.0101)^33^ with the following modifications: For Cor a 1.0101, streptomycin sulfate was added to a final concentration of 1% to precipitate nucleic acids. 1.5 M ﻿(NH_4_)_2_SO_4_ was then added to the protein solution, and the solution was loaded on to a 5 ml octylsepharose column (Octylsepharose 4 Fast Flow; GE Healthcare, Munich, Germany) equilibrated with 10 mM sodium phosphate, pH 7.0, 1.5 M (NH_4_)_2_SO_4_, and eluted using a gradient from 0 to 60% elution buffer (10 mM sodium phosphate, pH 7.0) followed by a step to 100% elution buffer.

Cor a 1.0102, Cor a 1.0103, or Cor a 1.0104 containing cell extracts were centrifuged (19000 g, 30 min, 4 °C) and the pellet was resuspended in 50 mM sodium phosphate, pH 7.8, 500 mM NaCl and 8 M urea. The denatured protein was refolded by stepwise lowering the urea concentration during dialysis to 4 M, 2 M, 1 M for 1 h and dialysis for 4 h and over night in 10 mM sodium phosphate, pH 7.0.

To remove remaining contaminants identified by ^1^H, ^15^N HSQC spectra, purified Bet v 1.0101, was unfolded using 8 M urea, 50 mM sodium phosphate pH 7.0, 50 mM NaCl for 1.5 h at room temperature. After centrifugation of the sample in a Vivaspin concentrator 20 (MWCO 10 kDa, Sartorius Stedim Biotech, Göttingen, Germany), 20 ml of buffer without urea was added to the remaining solution. After centrifugation the procedure was repeated and the sample was then concentrated to 1 ml. The pure proteins were either shock frozen and stored at −80 °C or dialysed against Milli Q H_2_O, lyophilised and stored at 4 °C.

### Protein analysis

Standard methods were used to analyse purity (SDS-PAGE), oligomeric state (SEC) and structural integrity (1D-NMR, ^1^H-^15^N HSQC spectroscopy for the ^15^N labelled proteins) of all variants. The proteins were stored as described above.

### Purification of nCor a 1 from pollen of *Corylus avellana* and ligand isolation

nCor a 1 was purified from hazel pollen (Allergom, Ängelholm, Sweden) as described previously for the isolation of Bet v 1 from birch pollen^33,36^ with minor changes, using ammonium sulfate precipitation (50%, 60% and 100% (NH_4_)_2_SO_4_ saturation), followed by hydrophobic interaction chromatography (HIC) as described above for the purification of recombinant Cor a 1.0101 but with only one purification step with 100% elution buffer. To remove remaining impurities, Cor a 1-containing HIC-fractions were concentrated (Vivaspin 20 concentrator, molecular-mass cut-off 3000 Da) to a final volume of 500 µl and loaded on to two consecutively connected Superdex 75 10/300 GL columns (24 ml bed volume each; GE Healthcare, Penzberg, Germany), equilibrated with a buffer containing 10 mM sodium phosphate pH 7.0, and 300 mM NaCl. If required, another HIC chromatography using a step gradient was performed. The nCor a 1 fractions were pooled, concentrated and lyophilised.

Subsequently, the prominent protein band at 17 kDa on an SDS polyacrylamide gel was excised and the protein was analysed by tryptic digestion followed by liquid chromatography – mass spectrometry (LC-MS^E^) to confirm its identity.

For ligand isolation pure Cor a 1 fractions were lyophilised and redissolved in methanol. The methanol extract was dried *in vacuo*, redissolved in 50 µl 2,5% methanol and analyzed by RP- HPLC using a 150 × 4 mm 5 µm C18A vertex plus column with a pore size of 100 Å (Knauer, Berlin, Germany), equilibrated with solvent A (2% acetic acid) followed by a gradient from 10% to 90% solvent B (0,5% acetic acid, 50% acetonitrile) within 35 min, and analysed by mass spectrometry.

### Mass spectrometric confirmation of Cor a 1 isoallergens and variants

The isoallergen and variant composition of purified nCor a 1 and the identity of purified rCor a 1.0102, rCor a 1.0103 and rCor a 1.0401 were determined by nano-UPLC (ultra performance liquid chromatography) nano-ESI MS^E^ (electron spray ionisation mass spectrometry)^46^ after SDS-PAGE separation and in gel digestion^47^ as published previously^45^ using an in-house database consisting of reviewed entries of the UniProt database (as at January 2016) (Table 2). The isoallergen and variant composition of crude hazel pollen extract was determined by nano-UPLC nano ESI MS^E^ ^46^ after in solution tryptic digestion as published previously^24^, except that we used the above mentioned in-house database for analyses. Three different crude extracts were obtained by extracting proteins from hazel pollen following an extraction protocol applied previously on birch pollen^45^. Differing from this, three buffers were used. Sample (a): sodium phosphate buffer^33^, (b): 100 mM NH_4_HCO_3_-buffer^45^ and (c): 8 mM Tris and 10 mM (NH_4_)_2_B_10_O_16_*8H_2_O.

### Mass spectrometry of the ligand Q3O-(Glc)-Gal

For MS analysis, the purified ligand extracted from hazel pollen was loaded on a HPLC RP-C18 column (Phenomenex Inc. USA, Kinetex 5 µm EVO C18, 100 Å, 30 × 2.1 mm) which was connected to a Q Exactive mass spectrometer (Thermo Fisher Scientific GmbH, Bremen, Germany) with a hybrid quadrupole orbitrap mass analyzer (maximum mass range 50–6000 Da, resolution 140.000 @ m/z = 200), using a gradient from 20–95% acetonitrile within 10 min. Mass spectra were acquired after (positive mode) electrospray ionisation (ESI pos) in full scan mode (70–1050 amu) recording the TIC.

To confirm the presence of the ligand of the purified nCor a 1, the methanol extract of nCor a 1 after SEC was loaded onto a C18 column (Accucore RP-MS, 2.6 µm, 150 × 2.1 mm) connected to the Q Exactive mass spectrometer. Isocratic elution was performed with 50% acetonitrile within 20 min. Mass spectra were acquired as described above.

### NMR experiments

All NMR-experiments were performed at 298 K on Bruker Avance spectrometers with proton resonance frequencies of 600, 700, 900 and 1000 MHz, the latter three equipped with cryogenically cooled triple resonance probes.

#### NMR-spectroscopy of ligands

^1^H-NMR and ^13^C NMR spectroscopy of about 3 mM Q3O-(Glc)-Gal in d_6_-DMSO was performed at 600 MHz ^1^H frequency. Chemical shifts were referenced to tetramethylsilane. Data was processed using Topspin version 3.2 (Bruker, Karlsruhe, Germany).

#### Determination of the solution structure of Cor a 1.0401 and Bet v 1.0101

Resonance assignments were done with standard double- and triple-resonance through-bond correlation experiments. Threedimensional NMR experiments of Cor a 1.0401 were recorded using non-uniform sampling (NUS) with 25% data amount. NMR-spectra to assign chemical shifts were obtained with a 600 µM [^1^H, ^13^C, ^15^N] Cor a 1.0401 sample in 10 mM sodium phosphate, pH 7.0, 10% (v/v) D_2_O, 0.03% NaN_3_ and 2 mM DTT. Three-dimensional ^13^C and ^15^N edited nuclear Overhauser enhancement spectroscopy (NOESY) experiments (mixing times 120 ms) were recorded for derivation of distance restraints. NMR data were processed using in-house software and visualized with NMRViewJ (OneMoon Scientific, Inc.). Iterative soft thresholding was applied for processing NUS NMR experiments^48^.

NOESY cross peaks were classified according to their relative intensities and converted into distance restraints with upper limits of 3.0 Å (strong), 4.0 Å (medium), 5.0 Å(weak) and 6.0 Å (very weak). Dihedral restraints were taken from analysis of chemical shifts by the TALOS software package^49^. Structures were calculated using the programme XPLOR-NIH^50,51^. The 20 structures showing the lowest overall energy were analysed with XPLOR-NIH and PROCHECK-NMR^52^.

#### Binding experiments

To investigate the binding interface of Cor a 1.0401 upon binding of quercetin, quercetin was dissolved in DMSO and added in different concentrations to 60 µM^13^ C^15^ N Cor a 1.0401, up to an equimolar concentration. The DMSO concentration of the final NMR sample was always 2,2%. To identify chemical shift perturbations caused by DMSO, a sample was prepared with 60 µM^13^ C^15^ N Cor a 1.0401 and 2.2% DMSO. ^1^H, ^15^N HSQCs of each sample were recorded. Where binding in the fast exchange rate occurred, chemical shift perturbations (CSPs) resulting from ligand binding were calculated based on equation (1):

$${\rm{\Delta }}{\delta }_{norm}=\sqrt{{({\rm{\Delta }}{\delta }_{HN})}^{2}+{(0.1{\rm{\Delta }}{\delta }_{N})}^{2}}$$

∆δ_HN_ and ∆δ_N_, chemical shift differences of amide proton and nitrogen resonances, respectively, in ppm.

Where binding in the slow exchange rate occurred, the relative intensities of each signal were compared to the corresponding signal in the reference spectrum without ligand.

For titration of Q3O-(Glc)-Gal to Cor a 1.0401, a 9.9 mM stock solution of Q3O-(Glc)-Gal in 10 mM sodium phosphate, pH 7.0 was prepared and added stepwise to 100 µM of ^13^C, ^15^N Cor a 1.0401 up to an 8-fold molar excess. After each titration step, a ^1^H, ^15^N HSQC and a ^1^H, ^13^C HSQC spectrum was recorded.

Binding specificity of Bet v 1.0101, Cor a 1.0401, Cor a 1.0101 and Cor a 1.0104 was investigated by recording ^1^H, ^15^N HSQCs of 60 µM of the respective ^15^N-labelled protein in the absence and in the presence of a ten-fold molar excess of Q3OS or Q3O-(Glc)-Gal.

### Computational methods

Figures of protein structures were generated and homologous protein structures were aligned using the programme PyMOL Molecular graphics System, Version 1.5.0.4. Theoretical isoelectric points, extinction coefficients and molecular weight of the different PR-10 proteins were determined using the ProtParam Tool^53^. For the calculation of the cavity volumes, the programme CastP^54^ was used with default parameters. They were determined for every single structure of the NMR bundle and are given as means + − S.D. Multiple and pairwise sequence alignments were performed with ClustalOmega^55^ and EMBOSS Needle^56^.

### Data deposition

Coordinates and restraints for structure calculation of Cor a 1.0401 and Bet v 1.0101 were deposited in the Protein Data Bank (PDB) under the accession codes 6GQ9 and 6R3C, respectively. Chemical shift assignments were deposited in the BioMagResBank: accession numbers 34281(Cor a 1.0401) and 34383 (Bet v 1.0101).

## Supplementary information

Jacob_Supplement_SciRep-REVISED_final
