# Supplementary material for: Identification of a natural ligand of the hazel allergen Cor a 1
Source: Sci Rep. 2019 Jun 18;9:8714. doi: 10.1038/s41598-019-44999-2 (PMC6582051; doi:10.1038/s41598-019-44999-2)
Supplement: Supplementary file 1 — Jacob_Supplement_SciRep-REVISED_final [file 41598_2019_44999_MOESM1_ESM.docx]

Identification of a natural ligand of the hazel allergen Cor a 1

**Thessa Jacob^1^, Christian Seutter von Loetzen^1^, Andreas Reuter^3^, Ulrike Lacher^4^, Dirk Schiller^3^, Rainer Schobert^4^, Vera Mahler^3^, Stefan Vieths^3^, Paul Rösch^1,2^_,_ Kristian Schweimer^1^, Birgitta M. Wöhrl^1#^**

**
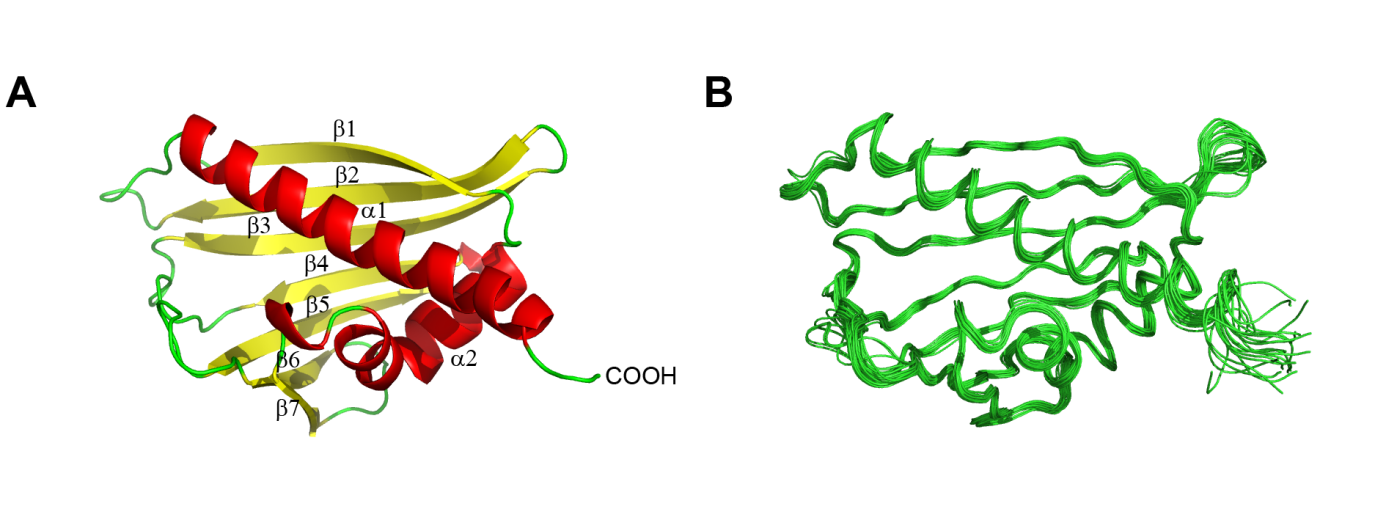
**

**Supplementary Figure S1: Solution structure of Bet v 1.0101. (A)** Cartoon representation of the average of the 20 lowest energy solution structures of Bet v 1.0101 (PDB: 6R3C). α-helices: red; β-strands, yellow; loop-regions, green. **(B)** Backbone overlay of the 20 lowest energy solution structures of Bet v 1.0101, with a backbone rmsd value of 0.46 Å and overall rmsd of 0.89 Å.


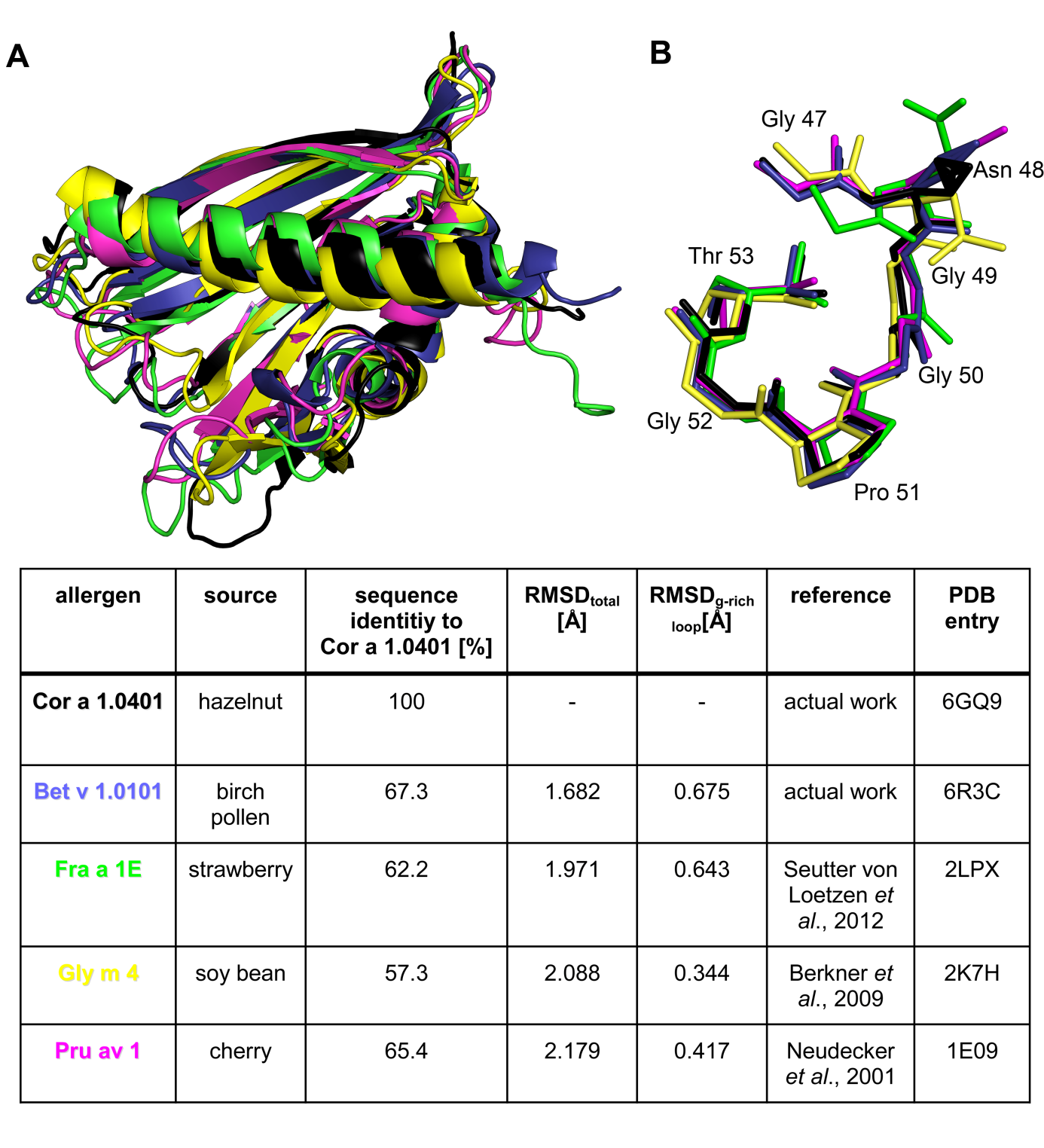


**Supplementary Figure S2: Structure of Cor a 1.0401 in comparison with homologous PR-10 allergens. (A)** Overlay of the cartoon representation of Cor a 1.0401 (PDB: 6GQ9, black) with Pru av 1 (1E09, pink), Bet v 1.0101 (6R3C, blue), Gly m 4 (2K7H, yellow) and Fra a 1E (2LPX, green). **(B)** Overlay of the glycine-rich loop (residues Gly 47 – Thr 53) in stick-representation. Colors as in **(A)**.


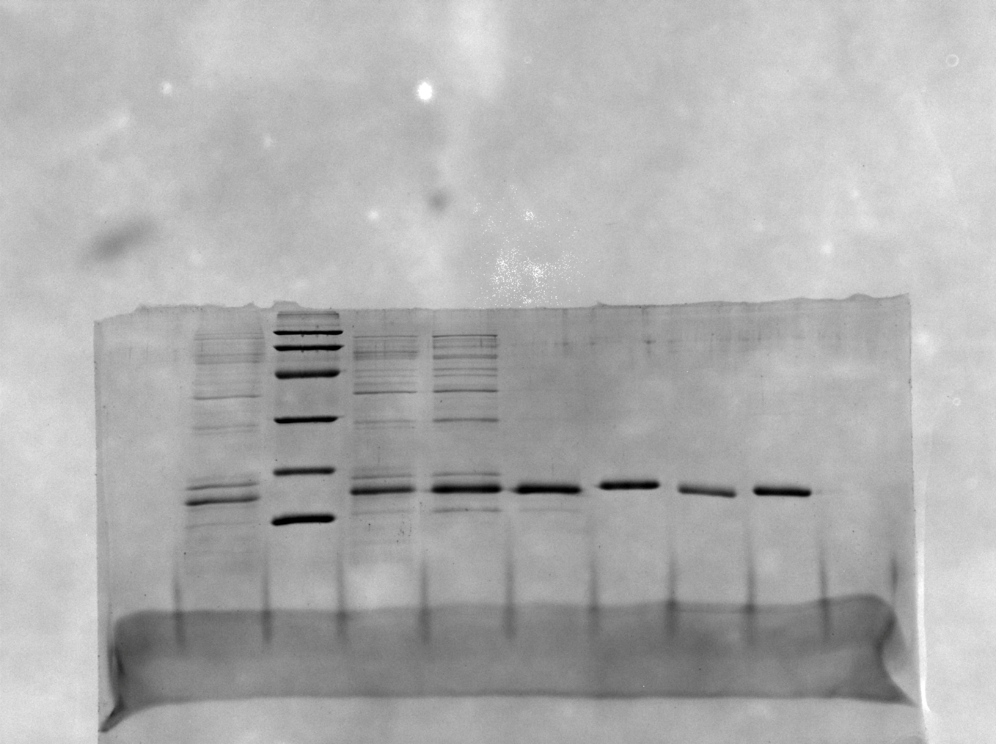


**Supplementary Figure S3: Purification of Cor a 1 from pollen**. Analysis of the purification procedure of Cor a 1 from hazel pollen by SDS-PAGE (19 % gel). (Uncropped gel from Fig. 2)


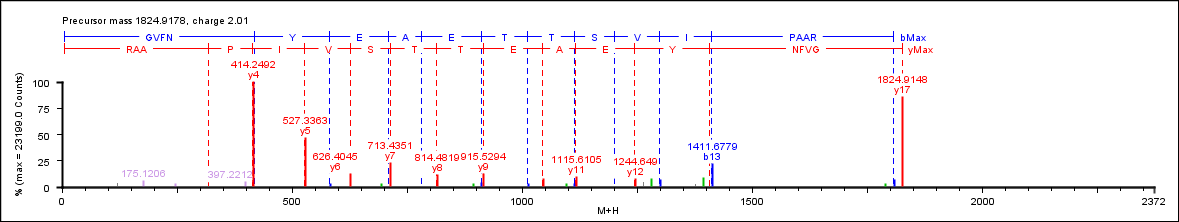


**A**


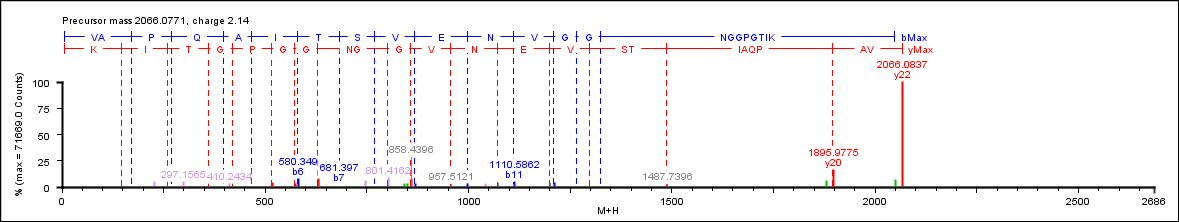


**B**

**Supplementary Figure S4: Annotated spectra of the variant specific peptides of Cor a 1.01 from natural purified Cor a 1.** Panel **A**: N-terminal peptide specific for Cor 1.0103. Panel **B**: Internal peptide specific for Cor a 1.0104.


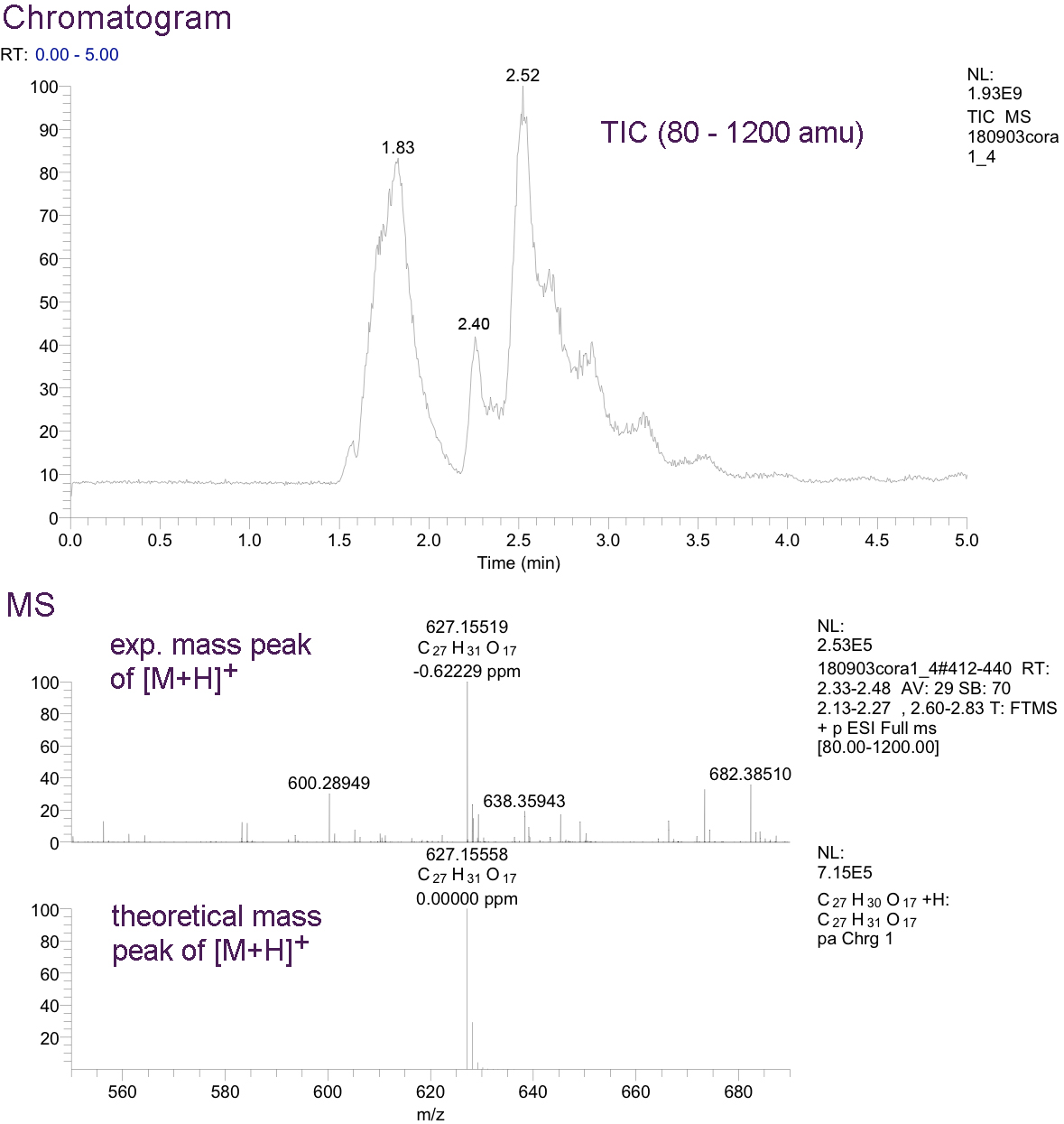


Supplementary Figure S5: Identification of Q3O-(Glc)-Gal extracted from nCor a 1. The upper panel shows the TIC of nCor a 1 MeOH extract. The second panel shows the mass spectrum at a retention time of 2.33 – 2.48 minutes with the experimental mass peak of 627.1552 m/z and the third panel the theoretical mass peak of Q3O-(Glc)-Gal [M+H]^+^ (627.1555 m/z).


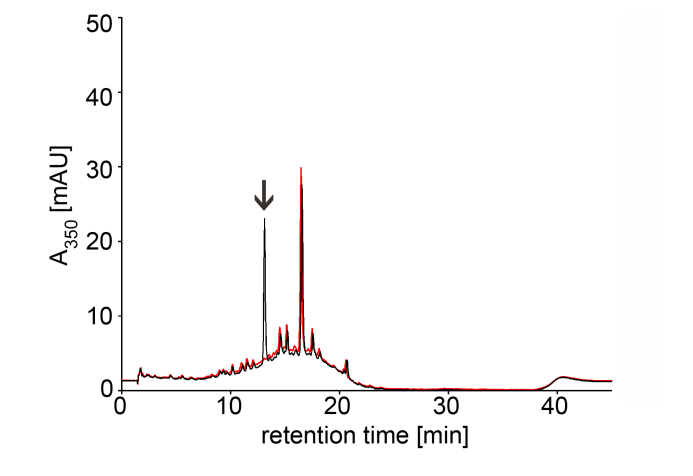


Supplementary Figure S6: HPLC chromatogram of hazelnut skin extracts.

Analysis of methanol extracts from hazelnut skin by RP-HPLC. Hazelnut skin extract before (red) and after the addition of 100 ng Q3O-(Glc)-Gal from hazel pollen (black). The additional peak in the black spectrum representing Q3O-(Glc)-Gal is indicated by an arrow.


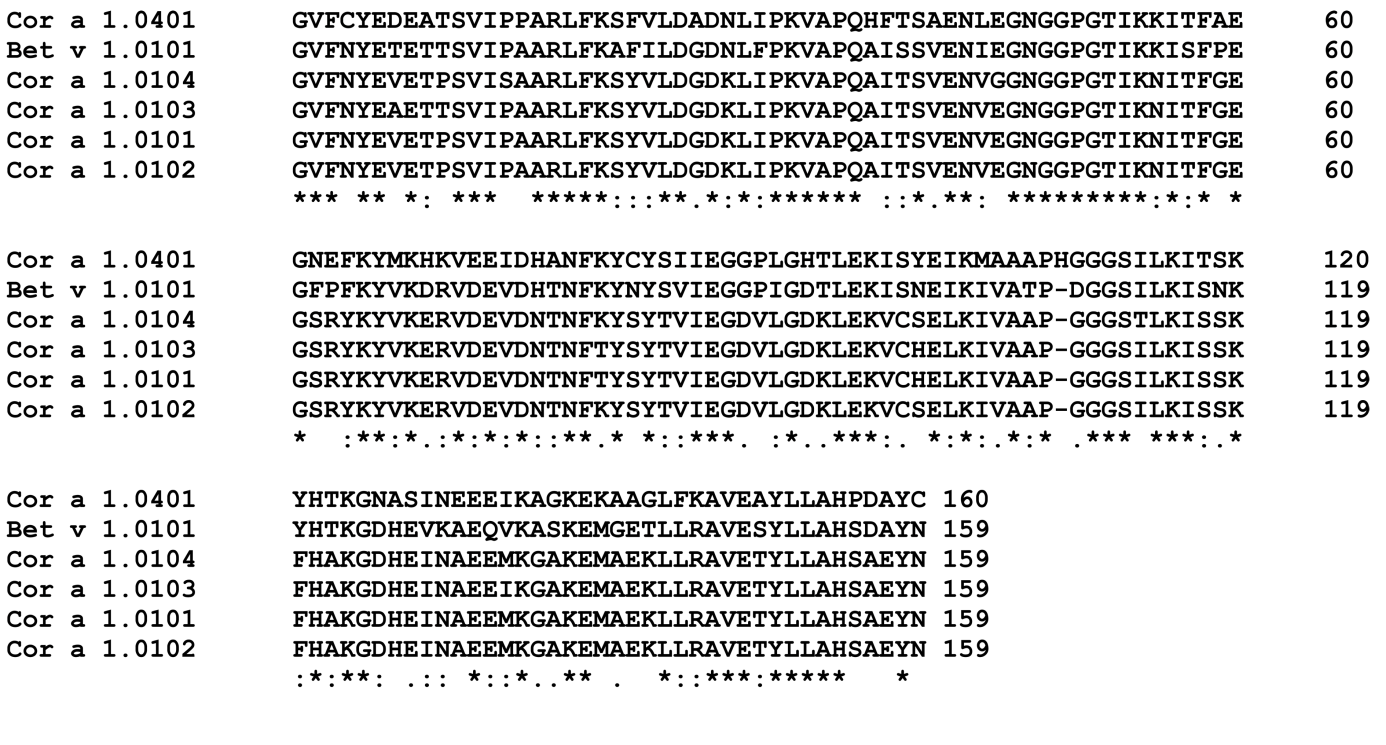


**Supplementary Figure S7: Amino acid sequence alignments of Cor a 1 and Bet v 1 proteins used**

**Supplementary Table S1: Solution structure statistics of Bet v 1.0101**

| **Experimentally derived restraints** |  |  |
| --- | --- | --- |
| distance restraints |  |  |
|  | NOE | 3643 |
|  | intraresidual | 1092 |
|  | sequential | 1001 |
|  | medium range | 572 |
|  | long range | 978 |
|  | hydrogen bonds | 64 |
|  |  |  |
| dihedral restraints |  | 212 |
|  |  |  |
| **restraint violation** |  |  |
| average distance restraint violation (Å) | 0.004798 +/- 0.000564 |  |
| distance restraint violation > 0.1 Å | 3.15 +/- 1.24 |  |
| average dihedral restraint violation (°) | 0.0561 +/- 0.0162 |  |
| dihedral restraint violation > 1° | 0.0 +/- 0.0 |  |
|  |  |  |
| **deviation from ideal geometry** |  |  |
| bond length (Å) | 0.000576 +/- 0.000034 |  |
| bond angle (°) | 0.1068 +/- 0.0048 |  |
|  |  |  |
| **coordinate precision^a,b^** |  |  |
| backbone heavy atoms (Å) | 0.46 |  |
| all heavy atoms (Å) | 0.89 |  |
|  |  |  |
| **Ramachandran plot statistics^c^** (%) | 92.7/7.0/0.3/0.0 |  |

^a^ The precision of the coordinates is defined as the average atomic root mean square difference between the accepted simulated annealing structures and the corresponding mean structure calculated for the given sequence regions.

^b^ calculated for residues 2-154

^c^ Ramachandran plot statistics are determined by PROCHECK ^52^ and noted by most favored/additionally allowed/generously allowed/disallowed.

**Supplementary Table S2: Summary of MS data on specific peptides of Cor a 1.01 isoforms variants**

| **PM** | **PE** | **S** | **Peptide Amino Acid Sequence** | **F** | **B- and Y- Fragments** | **FE** |
| --- | --- | --- | --- | --- | --- | --- |
|  |  |  |  |  |  |  |
| **Cor a 1.0103** |  |  |  |  |  |  |
| 1824.9178 | 2.9 | 8.3 | GVFNYEAETTSVIPAAR | 39 | b4b5b5*b6b6*b6°b7b7*b7°b8b8*b8°b9b9°b10b10°b11  b11°b12b12°b13b13°b17b17°y3y4y5y6y7y8y9y10y10°y1  1y11°y12y13y15°y17 | 9.2 |
|  |  |  |  |  |  |  |
| **Cor a 1.0104** |  |  |  |  |  |  |
| 2066.0771 | -5.1 | 8.4 | VAPQAITSVENVGGNGGPGTIK | 53 | b2b3b4b5b5*b6b6*b7b7°b8b8°b9b9°b10b10°b11b11°b  12b12°b13b13*b14b22*y1y2y3y4y5y5°y6y6°y7y7°y8*y9  y9*y9°y10y10*y10°y11y11*y12y12*y13y13*y14y16y16*  y20y20*y22y22* | 7.6 |
|  |  |  |  |  |  |  |
| PM: precursor mass in dalton; PE: precursor mass erreor in ppm; S: PLGS peptide score; F: number of y-series and b-series fragments; B- and Y-Fragments: specific y- and b- series fragments matched; FE: mean fragment mass error in ppm | | | | | | |
|  |  |  |  |  |  |  |

**Supplementary Table S3: Summary of MS data on unpurified extract from hazel pollen.**

| **internal** | **Genebank** | **UniProt** |  | **Description** |  |  | **S** |  |  | **SC** | **E** |  | **DP** | **MP** |
| --- | --- | --- | --- | --- | --- | --- | --- | --- | --- | --- | --- | --- | --- | --- |
|  |  |  |  |  |  |  |  |  |  |  |  |  |  |  |
| **crude extract buffer a** | |  |  |  |  |  |  |  |  |  |  |  |  |  |
| PEI127 | X70998 | Q08407 | Cora_Cora1 | Cor a 1 0104, Corylus avellana |  |  | 13119 |  |  | 62.9 | 5.8 |  | 12 | 1 |
| PEI126 | X70997 | Q08407 | Cora_Cora1 | Cor a 1 0103, Corylus avellana |  |  | 6270 |  |  | 58.5 | 6.1 |  | 10 | 0 |
| n.a. | n.a. | A4KA45 | PROF4_CORAV | Profilin 4, Corylus avellana |  |  | 7259 |  |  | 42.1 | 4.1 |  | 5 | 2 |
| n.a. | n.a. | O65002 | O65002_BETPN | Isoflavone reductase homolog Bet v 6 0101 Fragment |  |  | 599 |  |  | 14.3 | 4.3 |  | 6 | 0 |
| n.a. | n.a. | P21568 | CYPH_SOLLC | Peptidyl prolyl cis trans isomerase, Solanum lycopersicum |  |  | 481 |  |  | 23.4 | 16.2 |  | 7 | 3 |
| n.a. | n.a. | P34887 | CYPH_ALLCE | Peptidyl prolyl cis trans isomerase, Allium cepa |  |  | 468 |  |  | 22.0 | 6.4 |  | 3 | 1 |
| n.a. | n.a. | A2XGP6 | SODC1_ORYSI | Superoxide dismutase Cu Zn 1, Oryza sativa subsp indica |  |  | 413 |  |  | 11.2 | 16.8 |  | 2 | 0 |
| n.a. | n.a. | Q38936 | FK152_ARATH | Peptidyl prolyl cis trans isomerase FKBP15 2, Arabidopsis thaliana |  |  | 345 |  |  | 22.7 | 5.8 |  | 3 | 0 |
| n.a. | n.a. | P42739 | UBIQP_ACEPE | Polyubiquitin Fragment, Acetabularia peniculus |  |  | 203 |  |  | 8.0 | 6.8 |  | 4 | 0 |
| n.a. | n.a. | P29409 |  | Phosphoglycerate kinase chloroplastic Fragment, Spinacia oleracea |  |  | 175 |  |  | 8.5 | 23.2 |  | 3 | 0 |
|  |  |  |  |  |  |  |  |  |  |  |  |  |  |  |
| **crude extract buffer b** | |  |  |  |  |  |  |  |  |  |  |  |  |  |
| PEI127 | X70998 | Q08407 | Cora_Cora1 | Cor a 1 0103, Corylus avellana |  |  | 10884 |  |  | 57.2 | 7.3 |  | 8 | 0 |
| PEI126 | X70997 | Q08407 | Cora_Cora1 | Cor a 1 0104, Corylus avellana |  |  | 10199 |  |  | 62.9 | 7.1 |  | 11 | 1 |
| n.a. | n.a. | A4KA45 | PROF4_CORAV | Profilin 4, Corylus avellana |  |  | 15652 |  |  | 47.4 | 5.8 |  | 6 | 1 |
| n.a. | n.a. | P25816 | PROF1_BETPN | Profilin 1, Betula pendula |  |  | 13179 |  |  | 33.1 | 5.8 |  | 5 | 1 |
| n.a. | n.a. | A2XKU9 | COSA_ORYSI | Costars family protein, Oryza sativa |  |  | 2081 |  |  | 14.9 | 1.7 |  | 1 | 0 |
| n.a. | n.a. | O65002 | O65002_BETPN | Isoflavone reductase homolog Bet v 6 0101 Fragment, Betula pendula |  |  | 1756 |  |  | 24.7 | 5.3 |  | 8 | 0 |
| n.a. | n.a. | Q8L5T1 | Q8L5T1_BETPN | Peptidyl prolyl cis trans isomerase, Betula pendula |  |  | 1202 |  |  | 42.8 | 8.2 |  | 7 | 1 |
| n.a. | n.a. | Q38936 | FK152_ARATH | Peptidyl prolyl cis trans isomerase FKBP15 2, Arabidopsis thaliana |  |  | 1201 |  |  | 8.6 | 0.0 |  | 1 | 0 |
| n.a. | n.a. | P86354 | VIRE2_HELVI | Viresin Fragment, Heliothis virescens |  |  | 838 |  |  | 31.8 | 12.2 |  | 1 | 0 |
| n.a. | n.a. | P52578 | IFRH_SOLTU | Isoflavone reductase homolog, Solanum tuberosum |  |  | 481 |  |  | 10.4 | 9.1 |  | 5 | 0 |
| n.a. | n.a. | P09678 | SODC_BRAOC | Superoxide dismutase Cu Zn, Brassica oleracea |  |  | 480 |  |  | 6.6 | 4.1 |  | 2 | 0 |
| n.a. | n.a. | A2WN93 | CALM1_ORYSI | Calmodulin 1, Oryza sativa subsp indica |  |  | 477 |  |  | 47.0 | 15.4 |  | 6 | 0 |
| n.a. | n.a. | A4KA41 | PROF5_CORAV | Profilin 5, Corylus avellana |  |  | 325 |  |  | 33.6 | 3.9 |  | 2 | 0 |
| n.a. | n.a. | P29409 | PGKH_SPIOL | Phosphoglycerate kinase chloroplastic Fragment, Spinacia oleracea |  |  | 283 |  |  | 8.5 | 17.0 |  | 3 | 0 |
| n.a. | n.a. | P35131 | UBC8_ARATH | Ubiquitin conjugating enzyme E2 8, Arabidopsis thaliana |  |  | 242 |  |  | 22.3 | 19.3 |  | 2 | 0 |
| n.a. | n.a. | P60399 | RSMH_SPIKU | Ribosomal RNA small subunit methyltransferase H, Spiroplasma kunkelii | | | 181 |  |  | 8.0 | 3.5 |  | 1 | 0 |
| n.a. | n.a. | P0C030 | RUB1_ORYSJ | Ubiquitin NEDD8 like protein RUB1,Oryza sativa |  |  | 177 |  |  | 18.3 | 4.8 |  | 3 | 0 |
| n.a. | n.a. | Q1QEI9 | UBIG_PSYCK | Ubiquinone biosynthesis O methyltransferase, Psychrobacter cryohalolentis | | | 134 |  |  | 13.2 | 13.0 |  | 2 | 0 |
| n.a. | n.a. | P37943 | LEPP_BACNA | Signal peptidase I P, Bacillus subtilis |  |  | 125 |  |  | 29.0 | 1.6 |  | 3 | 0 |
| n.a. | n.a. | P48495 | TPIS_PETHY | Triosephosphate isomerase cytosolic, Petunia hybrida |  |  | 110 |  |  | 15.4 | 7.7 |  | 2 | 0 |
| n.a. | n.a. | P57550 | PPID_BUCAI | Peptidyl prolyl cis trans isomerase D, Buchnera aphidicola |  |  | 65 |  |  | 3.5 | 8.7 |  | 4 | 1 |
|  |  |  |  |  |  |  |  |  |  |  |  |  |  |  |
| **crude extract buffer c** | |  |  |  |  |  |  |  |  |  |  |  |  |  |
| PEI127 | X70998 | Q08407 |  | Cor a 1 0104, Corylus avellana |  |  | 13181 |  |  | 70.4 | 6.6 |  | 12 | 1 |
| PEI126 | X70997 | Q08407 |  | Cor a 1 0103, Corylus avellana |  |  | 9470 |  |  | 52.8 | 7.1 |  | 8 | 0 |
| n.a. | n.a. | A4KA45 |  | Profilin 4, Corylus avellana |  |  | 8513 |  |  | 57.1 | 6.5 |  | 7 | 2 |
| n.a. | n.a. | P25816 |  | Profilin 1, Betula pendula |  |  | 7512 |  |  | 57.1 | 6.9 |  | 7 | 2 |
| n.a. | n.a. | A4KA45 |  | Profilin 4, Corylus avellana |  |  | 8513 |  |  | 57.1 | 6.5 |  | 7 | 2 |
| n.a. | n.a. | A2XKU9 |  | Costars family protein, Oryza sativa |  |  | 2077 |  |  | 23.0 | 10.7 |  | 2 | 0 |
| n.a. | n.a. | Q8L5T1 |  | Peptidyl prolyl cis trans isomerase, Betula pendula |  |  | 1157 |  |  | 38.7 | 7.5 |  | 6 | 1 |
| n.a. | n.a. | O49886 |  | Peptidyl prolyl cis trans isomerase, Lupinus luteus |  |  | 1128 |  |  | 26.2 | 11.6 |  | 6 | 0 |
| n.a. | n.a. | O65002 |  | Isoflavone reductase homolog Bet v 6 0101 Fragment, Betula pendula |  |  | 1081 |  |  | 29.3 | 6.6 |  | 8 | 1 |
| n.a. | n.a. | P86354 |  | Viresin Fragment, Heliothis virescens |  |  | 864 |  |  | 31.8 | 7.7 |  | 1 | 0 |
| n.a. | n.a. | A4KA41 |  | Profilin 5, Corylus avellana |  |  | 505 |  |  | 33.6 | 6.7 |  | 3 | 0 |
| n.a. | n.a. | P09678 |  | Superoxide dismutase Cu Zn, Brassica oleracea |  |  | 425 |  |  | 16.4 | 24.2 |  | 3 | 0 |
| n.a. | n.a. | P35131 |  | Ubiquitin conjugating enzyme E2 8, Arabidopsis thaliana |  |  | 394 |  |  | 29.7 | 25.1 |  | 3 | 0 |
| n.a. | n.a. | O31535 |  | Uncharacterized protein YetH, Bacillus subtilis |  |  | 320 |  |  | 18.3 | 7.4 |  | 3 | 0 |
| n.a. | n.a. | Q41188 |  | Cold shock protein 2, Arabidopsis thaliana |  |  | 311 |  |  | 22.7 | 7.8 |  | 2 | 1 |
| n.a. | n.a. | P0C030 |  | Ubiquitin NEDD8 like protein RUB1, Oryza sativa |  |  | 235 |  |  | 19.0 | 3.1 |  | 3 | 0 |
| n.a. | n.a. | A1USC7 |  | 50S ribosomal protein L7 L12, Bartonella bacilliformis |  |  | 159 |  |  | 22.0 | 7.8 |  | 3 | 0 |
| n.a. | n.a. | P49310 |  | Glycine rich RNA binding protein GRP1A, Sinapis alba |  |  | 156 |  |  | 13.9 | 3.8 |  | 2 | 0 |
| n.a. | n.a. | A1W5A4 |  | Thymidylate synthase, Acidovorax sp |  |  | 82 |  |  | 5.1 | 31.2 |  | 3 | 0 |
| n.a. | n.a. | Q1PER6 |  | L ascorbate peroxidase 2 cytosolic, Arabidopsis thaliana |  |  | 75 |  |  | 8.0 | 4.5 |  | 1 | 0 |
| n.a. | n.a. | Q5F3T9 |  | UDP glucose 6 dehydrogenase, Gallus gallus |  |  | 66 |  |  | 7.1 | 24.6 |  | 3 | 1 |
| n.a. | n.a. | A4R017 |  | Probable zinc metalloprotease MGG 02107, Magnaporthe oryzae |  |  | 64 |  |  | 3.8 | 18.7 |  | 5 | 0 |
| n.a. | n.a. | Q4JV77 |  | Protein RecA, Corynebacterium jeikeium |  |  | 60 |  |  | 3.2 | 4.8 |  | 3 | 0 |
|  |  |  |  |  |  |  |  |  |  |  |  |  |  |  |
| n.a. not applicable; S: score, SC: sequence coverage; E avarage precursor mass error; DP: number of detected tryptic peptides; MP: number of detected modified peptides. | | | | | | | | | | | | | | |

**Supplementary Table S4: Sequence identity matrix of recombinant allergens used in this work**

|  | Cor a 1.0401 | Bet v 1.0101 | Cor a 1.0104 | Cor a 1.0103 | Cor a 1.0101 | Cor a 1.0102 | Protein bank entry |
| --- | --- | --- | --- | --- | --- | --- | --- |
| Cor a 1.0401 | 100.00 | 67.30 | 62.26 | 64.78 | 63.52 | 64.15 | AAD48405 |
| Bet v 1.0101 | 67.30 | 100.00 | 71.07 | 72.96 | 72.33 | 72.96 | CAA33887 |
| Cor a 1.0104 | 62.26 | 71.07 | 100.00 | 94.97 | 96.86 | 98.11 | CAA50326 |
| Cor a 1.0103 | 64.78 | 72.96 | 94.97 | 100.00 | 98.11 | 96.86 | CAA50325 |
| Cor a 1.0101 | 63.52 | 72.33 | 96.86 | 98.11 | 100.00 | 98.74 | CAA50327 |
| Cor a 1.0102 | 64.15 | 72.96 | 98.11 | 96.86 | 98.74 | 100.00 | CAA50328 |
